# Disassembly of All SNARE Complexes by *N*-Ethylmaleimide-sensitive Factor (NSF) Is Initiated by a Conserved 1:1 Interaction between α-Soluble NSF Attachment Protein (SNAP) and SNARE Complex*[Fn FN2]

**DOI:** 10.1074/jbc.M113.489807

**Published:** 2013-07-08

**Authors:** Sandro Vivona, Daniel J. Cipriano, Seán O'Leary, Ye Henry Li, Timothy D. Fenn, Axel T. Brunger

**Affiliations:** From the Departments of ‡ Molecular and Cellular Physiology,; § Structural Biology,; ¶ Photon Science,; ‖ Neurology and Neurological Sciences, and; ** Bioengineering and; the ‡‡ Howard Hughes Medical Institute, Stanford University Medical School, Stanford, California 94305

**Keywords:** ATPases, Biophysics, Biosensors, Circular Dichroism (CD), Enzyme Kinetics, Fluorescence, Membrane Fusion, Protein-Protein Interactions, SNARE Proteins, Structural Biology

## Abstract

Vesicle trafficking in eukaryotic cells is facilitated by SNARE-mediated membrane fusion. The ATPase NSF (*N*-ethylmaleimide-sensitive factor) and the adaptor protein α-SNAP (soluble NSF attachment protein) disassemble all SNARE complexes formed throughout different pathways, but the effect of SNARE sequence and domain variation on the poorly understood disassembly mechanism is unknown. By measuring SNARE-stimulated ATP hydrolysis rates, Michaelis-Menten constants for disassembly, and SNAP-SNARE binding constants for four different ternary SNARE complexes and one binary complex, we found a conserved mechanism, not influenced by N-terminal SNARE domains. α-SNAP and the ternary SNARE complex form a 1:1 complex as revealed by multiangle light scattering. We propose a model of NSF-mediated disassembly in which the reaction is initiated by a 1:1 interaction between α-SNAP and the ternary SNARE complex, followed by NSF binding. Subsequent additional α-SNAP binding events may occur as part of a processive disassembly mechanism.

## Teaser

**Background:** NSF and α-SNAP disassemble all SNARE complexes.

**Results:** The disassembly kinetics is conserved for different ternary and binary SNARE complexes. α-SNAP and the ternary SNARE complex form a 1:1 complex.

**Conclusion:** NSF uses a conserved mechanism to disassemble all SNARE complexes, starting from a 1:1 SNAP-SNARE complex interaction.

**Significance:** We illuminate a broad mechanism allowing NSF to support SNARE-mediated exocytosis.

## Introduction

The compartmentalization of eukaryotic cells (and the concomitant intracellular membrane trafficking) relies on a membrane fusion machinery that contains the so-called SNARE proteins. SNAREs contribute to membrane fusion specificity through the combination of different SNARE isoforms that are associated with different compartment membranes (1, 2), although other proteins also contribute to specificity (3). A characteristic feature of SNAREs is a core domain called the SNARE motif, which contains conserved heptad repeat patterns. The SNARE motifs contribute to a structurally conserved four-helix bundle in the so-called SNARE complex, which is formed by one v-SNARE (also called R-SNARE) and two or three t-SNAREs (also called Q-SNAREs) (4–6). Despite the structural conservation of the SNARE core complexes, there is considerable primary sequence variation in the core domains.

In addition to sequence variation, SNAREs show variability in their N-terminal domain architecture. Members of the syntaxin subfamily of t-SNAREs have an additional ∼25-residue long N-terminal peptide, followed by a three-helix bundle called the Habc domain (7). v-SNAREs, also called vesicle-associated membrane proteins (VAMPs),^2^ have a variety of N-terminal extensions: a 10-residue region in VAMP8/endobrevin; a proline-rich flexible 35-residue region in VAMP2/synaptobrevin-2; an ∼120-residue longin domain in VAMP7, Sec22b, and Ykt6 (8); and an ∼1000-residue WD40 domain in the VAMP homolog tomosyn (9). Despite the heterogeneity of SNARE complexes originating from the different isoforms, one molecular machine is capable of their disassembly: the AAA^+^ ATPase NSF (*N*-ethylmaleimide-sensitive factor) and its SNAP (soluble NSF attachment protein) adaptor protein (10–14). Unlike α-SNAP, the other two SNAP isoforms (β- and γ-SNAPs) are not ubiquitously expressed and will not be considered in this study.

The molecular details underlying the promiscuous recognition of different SNARE complexes by NSF and SNAP are poorly understood (15). Disassembly relies on the formation of a SNARE-SNAP-NSF complex called the 20S particle, followed by NSF-catalyzed ATP hydrolysis and the concomitant release of individual SNAREs. Based on biochemical and low-resolution cryo-EM reconstructions, it has been suggested that the 20S particle is composed of three α-SNAPs bound to a single ternary neuronal SNARE complex and that α-SNAP mediates the attachment of the toroidal NSF hexamer to the N-terminal side of the SNARE bundle (16–18). However, a few aspects of this model are unclear. The N-terminal domains of syntaxins (Habc domains) and some VAMPs (*e.g.* longin domains) are not accounted for. Furthermore, Marz *et al.* (17) identified one interaction interface on α-SNAP by mutagenesis studies, but it is unclear how this α-SNAP interface could bind three different interfaces on the asymmetric surface of the SNARE complex. Here, we performed comparative studies of binding constants and disassembly kinetics of four different physiological ternary SNARE complexes and one binary (t-SNARE) complex with NSF and α-SNAP. We found that the disassembly kinetics and binding properties are conserved for the four SNARE complexes and the binary complex and that α-SNAP and the SNARE complex initially interact with a 1:1 stoichiometry. We propose a conserved SNARE complex disassembly mechanism in which one α-SNAP binds the SNARE complex at any given time, with subsequent α-SNAP binding events possibly occurring during the disassembly process.

## EXPERIMENTAL PROCEDURES

### 

#### 

##### cDNA Constructs for Bacterial Expression

All DNAs used in this study encode rat proteins except NSF, which is from Chinese hamster. The recombinant expression of NSF and α-SNAP and the coexpression (*Escherichia coli*) of VAMP2(1–96) (simply referred to as VAMP2), syntaxin1A(1–265)-C145S/S249C/K253C (simply referred to as syntaxin1), syntaxin1A(191–265)-C145S/S249C/K253C (simply referred to as ^ΔN^syntaxin1), and SNAP25A (simply referred to as SNAP25) were performed as described (19). For coexpression of VAMP7 and VAMP8 with their corresponding SNAREs, the DNAs encoding VAMP7(1–190)-C21S/C56V (simply referred to as VAMP7), VAMP7(119–190) (simply referred to as ^ΔN^VAMP7), and VAMP8(1–76)-I76C (simply referred to as VAMP8) were fused to a C-terminal hexahistidine tag and cloned (NcoI/BamHI) in the pACYCDuet vector (Novagen) from commercially available, full-length open reading frames (GeneArt, Invitrogen) using standard PCR-based techniques. The tag-free syntaxin4A(1–274)-C141S/E257C/I261C (simply referred to as syntaxin4) and SNAP23 constructs were cloned into the first cloning site (BamHI/SalI) and the second cloning site (NdeI/XhoI) of the pETDuet vector (Novagen), respectively. Coexpression of the corresponding SNARE complexes (*i.e.* VAMP7-syntaxin1-SNAP25, VAMP7-syntaxin4-SNAP23, VAMP8-syntaxin4-SNAP23) was performed by transforming one pACYCDuet vector with the respective pETDuet vectors into competent cells. Expression of the syntaxin1-SNAP25 binary complex was achieved by transforming competent cells with one pACYCDuet vector containing syntaxin1A(1–265)-C145S/S249C/K253C and SNAP25A (cloned into NcoI/SalI and NdeI/XhoI, respectively). Disassembly of the VAMP7-syntaxin1-SNAP25 SNARE complex fused to either an N-terminal or a C-terminal His_6_ tag was compared to rule out a possible effect of the His_6_ tag on the disassembly reaction (data not shown).

##### Protein Expression and Purification

NSF was expressed and purified as described (19). All other recombinant proteins were expressed in BL21(DE3) cells (Invitrogen) at an absorbance of 0.6 by induction with 0.5 mm isopropyl 1-thio-β-d-galactopyranoside. The four different ternary SNARE complexes and the syntaxin1-SNAP25 binary complex were coexpressed for 12 h at 25 °C, whereas α-SNAP was expressed for 3 h at 37 °C. Cells were lysed by sonication after 30 min of incubation in buffer A (50 mm Tris-HCl (pH 8.0), 500 mm NaCl, and 1 mm dithiothreitol) with 10 mm imidazole, 0.5 mg/ml lysozyme, 5 μg/ml DNase, and protease inhibitors. Crude lysates were centrifuged for 40 min at 48,400 × *g*, and the supernatant was incubated with Ni^2+^-nitrilotriacetic acid-agarose beads (Qiagen) for 1 h at 4 °C. The beads were washed with buffer A with 30 mm imidazole and 0.1 mm tris(2-carboxyethyl)phosphine (TCEP; 50 column volumes). Beads loaded with α-SNAP-His_6_ were eluted with buffer A, 300 mm imidazole, and 0.1 mm TCEP (5 column volumes), whereas beads loaded with the SNARE complex were further washed (50 volume columns) and resuspended (1 volume column) in labeling buffer (20 mm HEPES (pH 7.4), 300 mm NaCl, 0.1 μm TCEP) for on-bead labeling with Oregon Green 488 (Invitrogen) for 15 min at 23 °C, washed again to remove excess dye, and finally eluted in buffer A with 300 mm imidazole and 0.1 mm TCEP (3 column volumes). The same protocol was used to biotinylate the VAMP2-syntaxin1-SNAP25 SNARE complex through use of maleimide-PEG2-biotin (Thermo Scientific). The His_6_ tag was removed from α-SNAP with tobacco etch virus protease (Invitrogen) by overnight dialysis (3500 nominal molecular weight cutoff, Pierce) at 4 °C. Cell pellets overexpressing the syntaxin1-SNAP25 binary complex were lysed by sonication after 30 min of incubation in 50 mm Tris-HCl (pH 8.0), 200 mm NaCl, 1 mm TCEP, 0.5 mg/ml lysozyme, 5 μg/ml DNase, and protease inhibitors. Crude lysates were centrifuged for 40 min at 48,400 × *g*, and the supernatant was loaded onto a Mono Q 10/100 anion exchange column (GE Healthcare) and eluted with a 0–100% gradient of 50 mm Tris-HCl (pH 8.0), 1 m NaCl, and 1 mm TCEP implemented over 20 column volumes. The binary complex dissociated from the column at ∼750 mm NaCl and was then gel-filtered in degassed labeling buffer to perform in-solution labeling with Oregon Green 488 dye for 15 min at 23 °C. Excess dye was removed by dialysis for 3 h at 23 °C (10 nominal molecular weight cutoff, Pierce). All protein preparations were finally purified by gel filtration on a Superdex 200 column (GE Healthcare) in 20 mm Tris-HCl (pH 8.0), 50 mm NaCl, and 0.1 mm TCEP. The progress and quality of the purification process were monitored by SDS-PAGE, mass spectrometry, CD spectroscopy, and multiangle light scattering (MALS).

##### ATPase Activity and SNARE Complex Disassembly Assays

NSF ATPase activity was measured using a microplate photometric assay coupled with the conversion of NADH (measuring absorbance at 340 nm) into NAD^+^ through pyruvate kinase and lactate dehydrogenase. All experiments were conducted simultaneously on a 96-well plate (150-μl samples, 0.5-cm path length) at 37 °C using a SpectraMax M2 plate reader (Molecular Devices). 20 nm NSF was added to 200 nm SNARE complex and 2 μm α-SNAP in disassembly buffer (50 mm Tris (pH 8.0), 20 mm NaCl, 0.1 mm TCEP, 0.5 mm ATP, 1 mm MgCl_2_).

The rates of NSF-driven disassembly of SNARE complexes were measured using the fluorescence dequenching method described previously (19) at 37 °C. Disassembly rates as a function of SNARE complex concentration were measured on a 384-well plate using a temperature-controlled FlexStation 3 plate reader (Molecular Devices). After base-line recording, 1.5 nm NSF was added to 30, 60, 100, 150, and 210 nm SNARE complex and a 12-fold α-SNAP-SNARE complex molar excess in disassembly buffer. Disassembly rates as a function of α-SNAP concentration were measured on a Hitachi F-4500 fluorometer (four ternary SNARE complexes) and on a SpectraMax M2 plate reader (binary SNARE complex). After base-line recording, 22 nm NSF was added to 220 nm SNARE complex and 0, 0.1, 0.2, 0.3, 0.4, 0.8, 1.7, 3.3, and 7.5 μm α-SNAP.

##### MALS

Size exclusion chromatography (SEC) coupled with MALS (SEC-MALS) was performed by injecting 200 μl of the following samples onto a WTC-030S5 column (Wyatt Technology) at a flow rate of 0.5 ml/min: (i) 30 μm ^ΔN^VAMP7-^ΔN^syntaxin1-SNAP25, (ii) 30 μm α-SNAP, (iii) 30 μm ^ΔN^VAMP7-^ΔN^syntaxin1-SNAP25 and 30 μm α-SNAP, (iv) 30 μm ^ΔN^VAMP7-^ΔN^syntaxin1-SNAP25 and 90 μm α-SNAP, and (v) 30 μm VAMP2-syntaxin1-SNAP25 and 90 μm α-SNAP. Measurements were performed in 20 mm Tris-HCl (pH 8.0), 50 mm NaCl, and 0.1 mm TCEP. The elution profile was monitored by UV absorption at 280 nm (Jasco UV-975 UV-visible system), light scattering at 658 nm (HELEOS system, Wyatt Technology), and differential refractometry (Optilab system, Wyatt Technology). Data analyses were carried out using ASTRA 6.0 software (Wyatt Technology). A differential refractive index increment (*dn*/*dc*) value of 0.185 was used in all calculations.

##### Biolayer Interferometry

Analyses were conducted at 30 °C on an Octet RED system (FortéBio) using 96-well microplates (Greiner Bio-One) in 20 mm Tris-HCl (pH 8.0), 20 mm NaCl, 0.1 mm TCEP, 0.1 mg/ml BSA, and 0.002% Tween 20. Streptavidin-coated tips were loaded over 200 s in 80 nm SNARE complex (VAMP2-syntaxin1-SNAP25) containing biotinylated cysteines with a capture level of ∼2.1 ± 0.2 nm within a row of eight tips. To monitor 7 S complex formation as a function of α-SNAP concentration, SNARE complex-loaded tips were dipped into an α-SNAP dilution series (0, 0.094, 0.188, 0.375, 0.75, 1.5, 3, and 6 μm) and allowed to associate for 100 s. Dissociation (100 s) was performed in buffer. A reference plate with no ligands was used to subtract unspecific binding.

## RESULTS

### 

#### 

##### Conservation of Disassembly Kinetics

**FIGURE 1.**
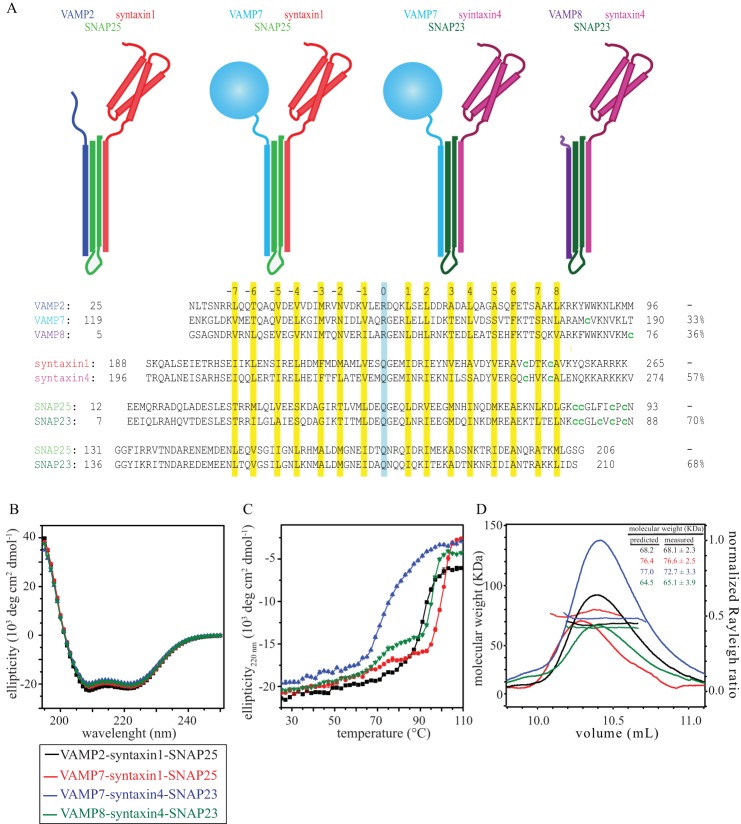
*A*, ternary SNARE complexes used in this study. *Upper*, schematic representation of the four ternary SNARE complexes used in this study. *Lower*, sequence alignment of the corresponding SNARE core domains. The heptad repeats and ionic layer are highlighted in *yellow* and *cyan*, respectively. The endogenous and mutant cysteines used for maleimide-Oregon Green 488 labeling are indicated (*lowercase green c*). The percentage of sequence identity between single SNARE domains is indicated on the right. *B*, CD of the four ternary SNARE complexes showed wavelength scans typical of α-helical structures, with characteristic minima at 208 and 220 nm and positive values below 200 nm. *C*, thermal melts of the four ternary SNARE complexes, monitored as loss of ellipticity at 220 nm *versus* temperature (25–110 °C), indicated high thermal stability. *D*, SEC-MALS experiments showed that the four ternary SNARE complexes are monomeric. The *inset* reports the predicted and measured molecular masses (in kDa) of the four ternary complexes. In the chromatogram, the *lines* report the molecular mass, and the *curves* report the light scattering (*i.e.* normalized Rayleigh ratio) as a function of elution volume on a WTC-100S5 column (Wyatt Technology). *deg*, degrees.

To compare the kinetics of NSF-driven disassembly of SNARE variants, we selected four different physiological SNARE complexes with differences in both primary sequence and domain architecture (Fig. 1*A*). VAMP2-syntaxin1-SNAP25 (VAMP2 is also referred to as synaptobrevin-2) is the neuronal SNARE complex involved in neurotransmitter release (2). VAMP7-syntaxin1-SNAP25 forms at the neuronal plasma membrane, where VAMP7 sustains axon outgrowth through transport of the cell adhesion molecule L1 (20, 21) and is thought to activate exocytosis of the resting pool of synaptic vesicles (22). VAMP7-syntaxin4-SNAP23 participates in synaptotagmin VII-regulated lysosomal exocytosis in fibroblasts (23). VAMP8-syntaxin4-SNAP23 is involved in cytokine/chemokine trafficking by segregating lysosomal secretory granules in mast cells (24). These four different ternary SNARE complexes allowed us to test if NSF-driven disassembly is influenced by primary sequence variation of the SNARE core domains and by differences in the N-terminal domains (*i.e.* VAMP2 *versus* VAMP7).

The four selected ternary SNARE complexes (Fig. 1*A*) were coexpressed, purified, and labeled as described previously (19) and as described under “Experimental Procedures.” Proper folding, thermal stability, and oligomeric state were assessed by CD spectroscopy (Fig. 1, *B* and *C*) and SEC-MALS (Fig. 1*D*).

**FIGURE 2.**
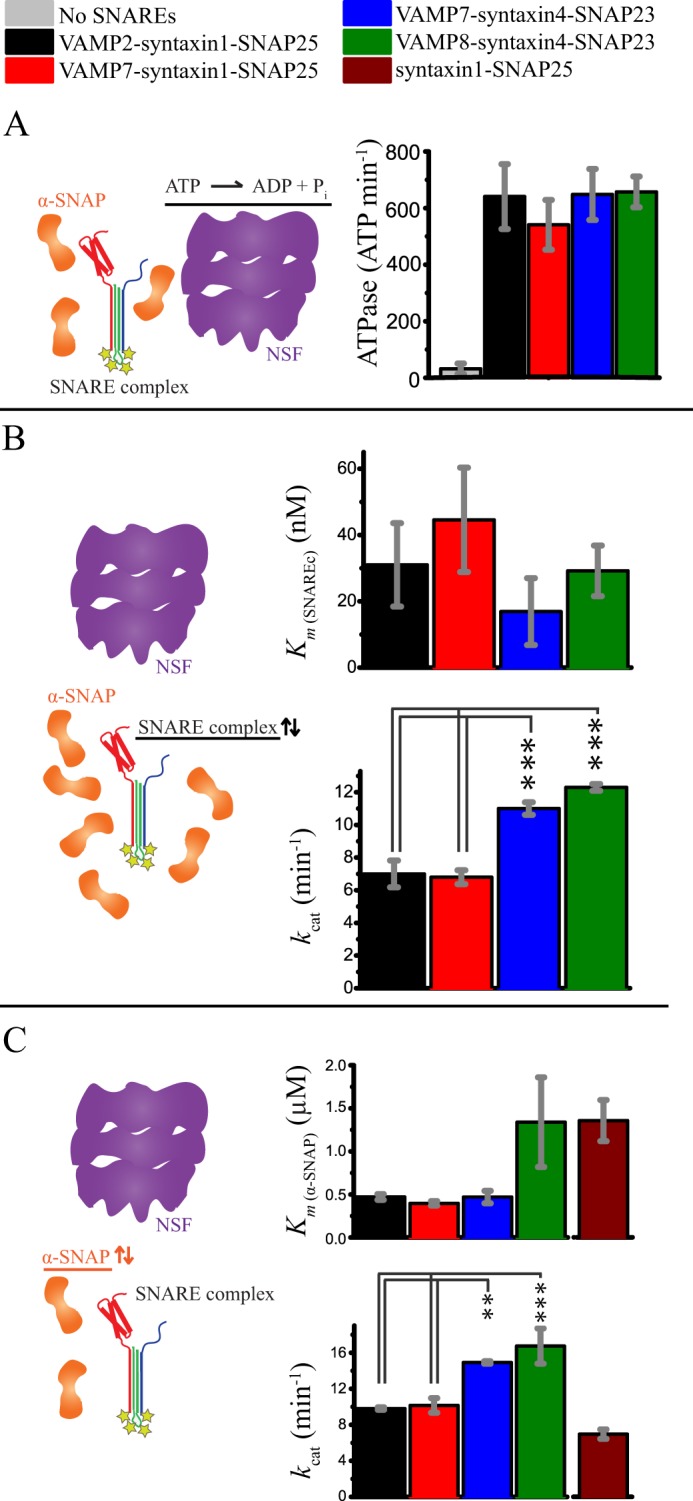
**Comparison of ATPase stimulation and disassembly kinetics for ternary and binary SNARE complexes.** All bar diagrams show means ± S.D. from three replicates. Statistical comparisons were by one-way analysis of variance with a Bonferroni post hoc test. **, *p* < 0.01; ***, *p* < 0.001. *A*, stimulation of ATPase activity by ternary SNARE complexes in the presence of α-SNAP. All four ternary SNARE complexes similarly stimulated the ATPase activity of NSF. *B*, steady-state kinetics of NSF-driven disassembly of the four ternary SNARE complexes. The means ± S.D. of *k*_cat_ and *K_m_* values were obtained from fitting the Michaelis-Menten equation (*y* = *k*_cat_·*x*/(*K_m_* + *x*)) to the observed initial disassembly rates as a function of SNARE complex concentration for three independent experiments (see also supplemental Fig. S1*A*). *K_m_* values are statistically similar for the four ternary complexes, although there was a small difference between the *k*_cat_ obtained for the complexes containing syntaxin1-SNAP25 and those containing syntaxin4-SNAP23. *C*, steady-state kinetics of NSF-driven disassembly for the four ternary SNARE complexes and the neuronal t-SNARE (syntaxin1-SNAP25) complex as a function of α-SNAP concentration. The means ± S.D. of *K_m_* and *k*_cat_ values were obtained by fitting the Michaelis-Menten equation to the observed initial disassembly rates as a function of α-SNAP concentration for three independent experiments (see also supplemental Fig. S1*B*). Similar to *B*, a small difference was observed between the *k*_cat_ of the complexes containing syntaxin1-SNAP25 and the *k*_cat_ of those containing syntaxin4-SNAP23. The slight variation of *k*_cat_ values in *B* and *C* is likely due to batch-to-batch variability of different NSF preparations and temperature variations across different instruments and experiments.

We measured the stimulation of NSF ATPase activity by the four different ternary SNARE complexes. All four complexes similarly stimulated the steady-state rate of NSF-catalyzed ATP hydrolysis activity (Fig. 2*A*), suggesting that the stimulation of ATPase activity is independent of SNARE primary sequence variation and N-terminal domain architecture.

We next investigated whether the similarity of ATPase activity stimulation would be reflected in the kinetics of the SNARE complex disassembly itself for the four ternary SNARE complexes. We studied the steady-state kinetics of NSF-driven disassembly of the four SNARE complexes by measuring the initial disassembly rates as a function of SNARE complex concentration (Fig. 2*B* and supplemental Fig. S1*A*) and fitted them to a first-order Michaelis-Menten equation (*y* = *k*_cat_·*x*/(*K_m_* + *x*)). We obtained statistically similar *K_m_* values, ranging from 17 to 45 nm (Fig. 2*B* and supplemental Fig. S1), implying similar apparent specificity of NSF for the four SNARE complexes. However, we found small differences between the *k*_cat_ of the two complexes containing the t-SNAREs syntaxin1 and SNAP25 and that of the two containing the t-SNAREs syntaxin4 and SNAP23 (Fig. 2*B*).

To test the requirement for α-SNAP in the disassembly of the four complexes, we also measured the initial disassembly rates as a function of α-SNAP concentration (Fig. 2*C* and supplemental Fig. S1*B*). The resulting plots were fitted with a simple first-order Michaelis-Menten equation (*y* = *k*_cat_·*x*/(*K_m_* + *x*)) and yielded *K_m_* values that are statistically similar across different SNARE complexes and consistent with the *K*_0.5_ values reported by Lauer *et al.* (25) for the VAMP2-syntaxin1-SNAP25 complex. In analogy to Fig. 2*B*, there is a small difference between the *k*_cat_ observed for the two complexes containing syntaxin4-SNAP23 and that observed for the two complexes containing syntaxin1-SNAP25.

Previous reports have shown qualitatively that α-SNAP can interact with isolated t-SNAREs, but not with isolated VAMPs (26, 27), and that α-SNAP-NSF can disassemble binary complexes containing t-SNAREs only (27–31). To quantitate these early findings, we compared the disassembly kinetics of binary (*i.e.* VAMP-free) and ternary complexes (Fig. 2*C* and supplemental Fig. S1*B*). This analysis yielded kinetic values that are statistically similar to those of the corresponding VAMP2/VAMP7-syntaxin1-SNAP25 ternary complexes (Fig. 2*C*). Taken together, the data presented in Fig. 2 suggest that NSF disassembles both ternary and binary SNARE complexes through a conserved mechanism, with a small dependence on the composition of the t-SNARE part of the SNARE complex.

##### Conserved Interaction between α-SNAP and the Ternary SNARE Complex

**FIGURE 3.**
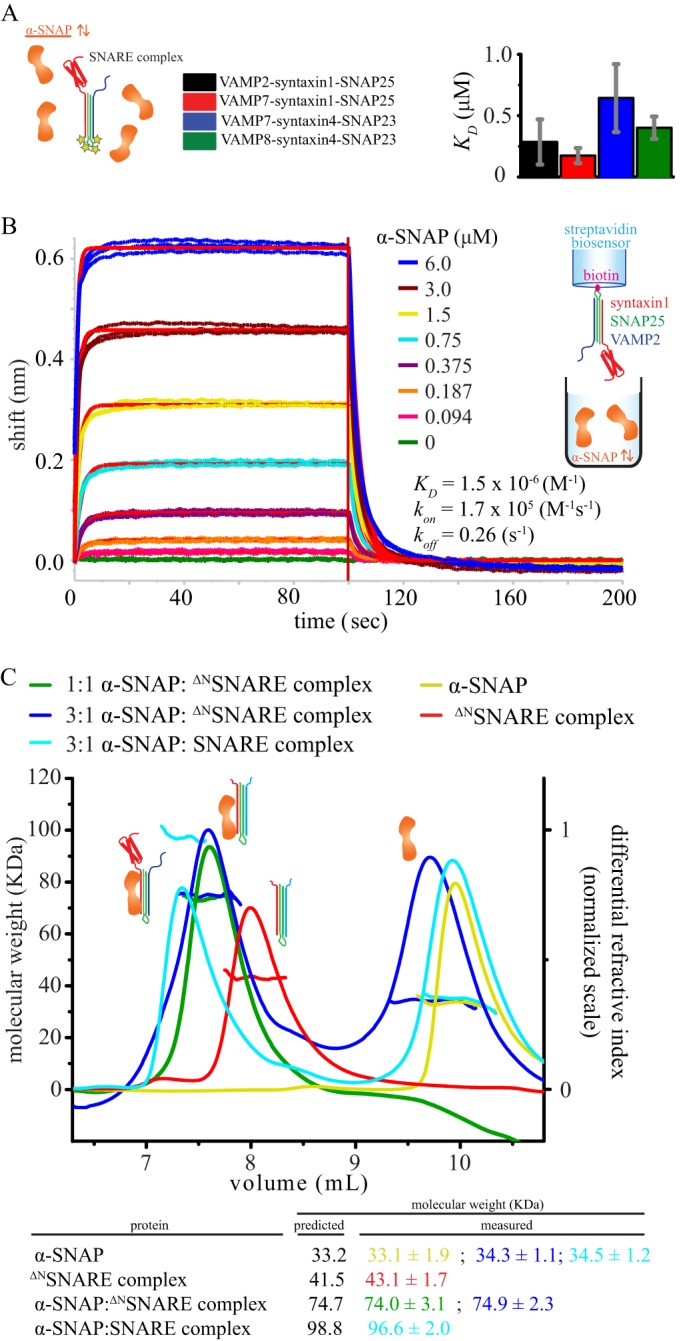
**α-SNAP-SNARE interaction and stoichiometry.** *A*, binding of α-SNAP to the four ternary SNARE complexes. The means ± S.D. of the equilibrium dissociation constant (*K_D_*) were obtained from fitting the equation *y* = *F*_max_·*x*/(*K_D_* + *x*) to the increase in basal fluorescence of the labeled SNARE complexes as a function of α-SNAP concentration for three independent experiments (see also supplemental Fig. S2). No significant difference (*p* < 0.01) was observed for the four SNARE complexes by one-way analysis of variance with a Bonferroni post hoc test. *B*, biolayer interferometry of α-SNAP interacting with biotinylated VAMP2-syntaxin1-SNAP25 loaded onto streptavidin sensors. Three replicates of association and dissociation phases are shown. One global fit was used to fit all of the data. *C*, *upper*, SEC-MALS of the ^ΔN^VAMP7-^ΔN^syntaxin1-SNAP25 core complex (referred to as the ^ΔN^SNARE complex), α-SNAP, 1:1 (*i.e.* 30 μm) and 1:3 mixtures of ^ΔN^SNARE and α-SNAP, and a 1:3 mixture of VAMP2-syntaxin1-SNAP25 (referred to as the SNARE complex) and α-SNAP. Molecular mass and normalized differential refractive index chromatograms of the four samples are overlaid and reported as a function of elution volume using a WTC-030S5 column (Wyatt Technology). *Lower*, the predicted and measured molecular masses (in kDa) are shown.

We hypothesized that our results could be explained by a conserved interaction between the adaptor protein α-SNAP and the SNARE complex. To test this hypothesis, we took advantage of the observation that the fluorescence quantum yield of complexes labeled at their C termini with Oregon Green is sensitive to α-SNAP binding (supplemental Fig. S2), likely due to the change of chemical environment sensed by the fluorophores upon interaction with α-SNAP. Measuring the change in Oregon Green fluorescence intensity as a function of α-SNAP concentration and fitting the resulting titration curves with a simple first-order binding model (*y* = *F*_max_·*x*/(*K_D_* + *x*)) yielded no significant differences among dissociation constants (*K_D_*) (Fig. 3*A* and supplemental Fig. S2), supporting an overall common mode of binding of α-SNAP to all SNARE complexes, in agreement with the similar steady-state kinetics of the disassembly reaction (Fig. 2). This effect is α-SNAP-specific, as no change is seen with BSA (supplemental Fig. S2). It is also in agreement with previous reports suggesting that α-SNAP binds to the C terminus of the SNARE complex (17, 26, 32).

To validate these results, we studied the α-SNAP-SNARE complex interaction using biolayer interferometry. Biotinylated SNARE complex (VAMP2-syntaxin1-SNAP25) was loaded onto streptavidin sensors and then dipped into α-SNAP solution at different concentrations (Fig. 3*B*). Complete dissociation was achieved upon buffer exchange. The experiment consisted of three replicates globally fit together with a simple 1:1 model to extract the kinetic parameters. The *K_D_* obtained with this analysis (*i.e.* 1.5 μm) agrees within ∼3-fold with the highest value allowed with our fluorescence-based binding constant (0.47 μm), a relatively small difference considering the differences in techniques.

##### α-SNAP Forms a 1:1 Complex with the Ternary SNARE Complex

Our fluorescence and biolayer interferometry data of α-SNAP binding to the four different SNARE complexes can be explained with a simple 1:1 binding model. However, more complicated models with multiple binding events could be possible as well. To determine the stoichiometry, we incubated α-SNAP and the core of the VAMP7-syntaxin1-SNAP25 SNARE complex (without the N-terminal domains; 30 μm each, corresponding to a saturating concentration), and we measured the molecular mass of the resulting complex by SEC-MALS. The mixture of α-SNAP and ^ΔN^VAMP7-^ΔN^syntaxin1-SNAP25 eluted as a single species with a molecular mass of 74.0 ± 3.1 kDa, which agrees well with a 1:1 complex (Fig. 3*C*). We then repeated the experiment with a 3-fold molar excess of α-SNAP (30 μm ^ΔN^VAMP7-^ΔN^syntaxin1-SNAP25 and 90 μm α-SNAP) and found that the excess of α-SNAP did not alter the stoichiometry of the complex of α-SNAP with ^ΔN^VAMP7-^ΔN^syntaxin1-SNAP25 and that the excess α-SNAP eluted later as monomeric isolated species (Fig. 3*C*). Similar results were obtained for the VAMP2-syntaxin1-SNAP25 complex (Fig. 3*C*). Taken together, our SEC-MALS results suggest a 1:1 stoichiometry for the complex between α-SNAP and the ternary SNARE complex.

##### Conservation of the Electrostatic Potential Surface of SNARE Complexes

**FIGURE 4.**
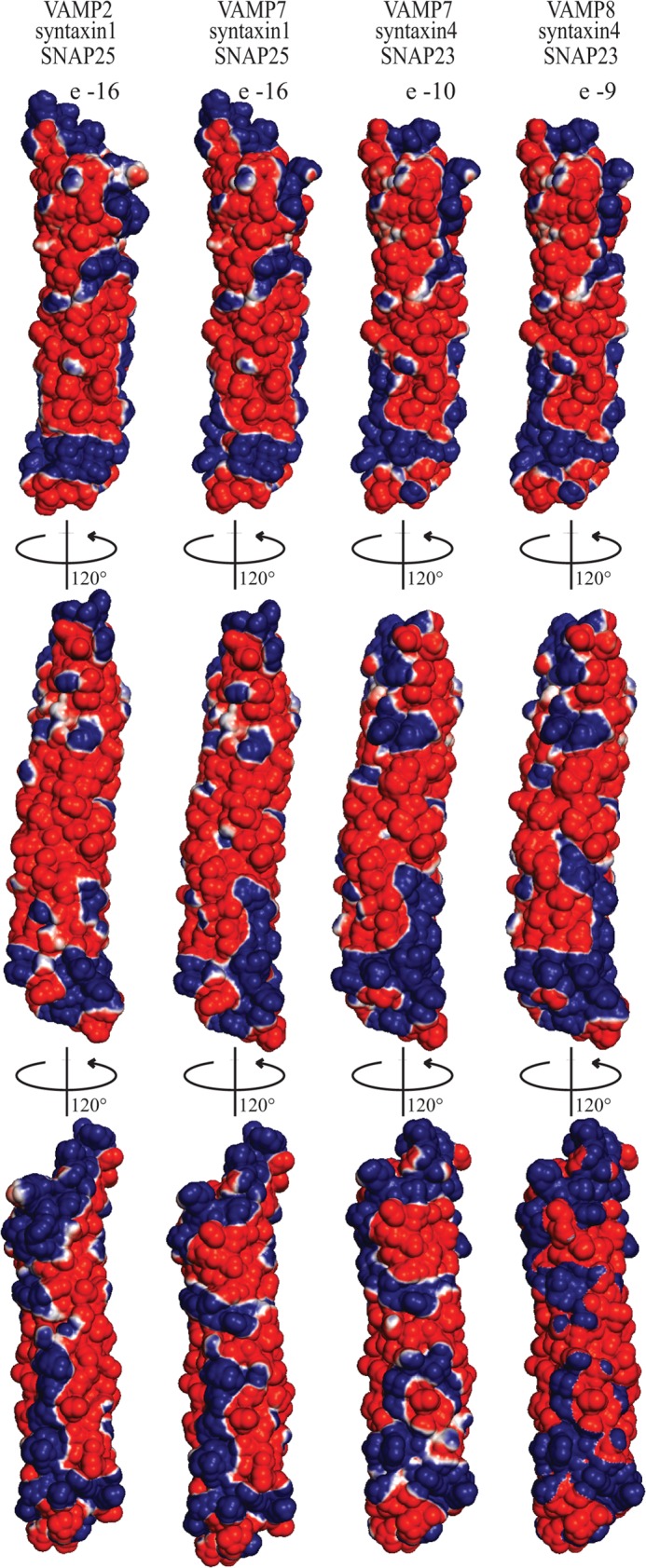
**Conservation of the electrostatic potential surface of ternary SNARE complexes.** *Upper*, electrostatic potential surface maps of (from *left* to *right*) the VAMP2-syntaxin1-SNAP25, VAMP7-syntaxin1-SNAP25, VAMP7-syntaxin4-SNAP23, and VAMP8-syntaxin4-SNAP23 core complexes (*i.e.* without the respective N-terminal domains). *Center*, 120° counterclockwise rotation of the top view. *Lower*, 120° counterclockwise rotation of the center view. The electrostatic potential surface maps were calculated in vacuum with PyMOL and contoured with the ABPS module from −10 *kT*/e (*red*) to +10 *kT*/e (*blue*). The SNARE core complexes of VAMP7-syntaxin1-SNAP25, VAMP7-syntaxin4-SNAP23, and VAMP7-syntaxin4-SNAP23 were modeled based on the crystal structure of VAMP2-syntaxin1-SNAP25 (Protein Data Bank code 1SFC).

Our kinetic and binding data suggest a conserved mechanism for NSF-driven disassembly of SNARE complexes, mediated by a conserved interaction between α-SNAP and both binary and ternary SNARE complexes. These common features may arise from a conserved electrostatic potential surface of the SNARE complex, based on the observation that the SNARE complex interacts with α-SNAP mostly through its acidic residues (17). Indeed, modeling the SNARE core bundles of VAMP7-syntaxin1-SNAP25, VAMP7-syntaxin4-SNAP23, and VAMP8-syntaxin4-SNAP23 based on the crystal structure of the neuronal SNARE complex (VAMP2-syntaxin1-SNAP25; Protein Data Bank Code 1SFC (4)) revealed a conserved electrostatic potential surface distribution (Fig. 4), suggesting that α-SNAP recognizes different SNARE complexes via conserved features of their electrostatic potential surfaces.

## DISCUSSION

### 

#### 

##### Conserved Disassembly Kinetics for Ternary and Binary SNARE Complexes

The ATPase NSF and its adaptor protein α-SNAP recognize and disassemble SNARE complexes in a promiscuous manner. The SNARE core domains show only limited amino acid sequence identity (for example, 33% identity between VAMP2 and VAMP7) (Fig. 1*A*), and the N-terminal domains can be quite different (Fig. 1*A*). The question arises at to how such promiscuous recognition is mediated in molecular detail. Moreover, the architecture of the 20S complex and the location of the SNARE N-terminal domains remain unclear, even in the recent low-resolution EM reconstruction (18).

Previous studies of the NSF molecular mechanism focused on the neuronal SNARE complex formed by VAMP2, syntaxin1, and SNAP25, so the possible effects of SNARE sequence variations and SNARE N-terminal domains on the disassembly had not been considered (25, 33, 34). Our study thus goes beyond this previous body of work by measuring the disassembly kinetics of four selected ternary SNARE complexes with both sequence and N-terminal domain variations. We found that NSF disassembles the selected four ternary SNARE complexes with similar rates and affinities (Fig. 2), suggesting a conserved molecular mechanism that is not influenced by the presence and type of SNARE N-terminal domains.

Our disassembly kinetics data revealed statistically similar *K_m_* values (Fig. 2*B*) and α-SNAP dependence for the disassembly of the different SNARE complexes (Fig. 2*C*). Moreover, the disassembly kinetics for the binary neuronal complex (t-SNAREs syntaxin1 and SNAP25) was similar to that for the corresponding ternary complexes (Fig. 2*C*).

##### A Conserved Interaction Site between α-SNAP and the SNARE Complex

Consistent with previous observations (27–31), our disassembly kinetics data suggest a conserved α-SNAP interaction with the ternary SNARE complex to initiate 20S complex formation. In particular, α-SNAP interacts with all four ternary SNARE complexes with statistically similar equilibrium dissociation constants (*K_D_* = 0.2–0.6 μm) (Fig. 3*A*) and produces similar *K_m_* values as observed by steady-state kinetics (Fig. 2*C*). Moreover, using SEC-MALS, we found that α-SNAP and the SNARE complex interact with a 1:1 stoichiometry (Fig. 3*B*). Our fluorescence, biolayer interferometry, and SEC-MALS data (Fig. 3) collectively point to an initial 1:1 α-SNAP-SNARE complex. It is possible that the affinity of this interaction *in vivo* could be different from what we observed in solution, as the capability of α-SNAP to bind membranes may favor the encounter with the SNARE complex (32).

Previous mutagenesis studies suggested that a mostly basic face of α-SNAP binds to the ternary neuronal SNARE complex (17). On the basis of this and conservation of the electrostatic potential surface among the four different SNARE complexes (Fig. 4), we speculate that one α-SNAP likely binds the four different ternary SNARE complexes through a negative electrostatic pattern produced by a conserved motif involving the t-SNARE components of the SNARE complexes. This reasoning is based on our observation that the disassembly kinetics of the binary (t-SNARE) complex is similar to that of the ternary SNARE complex.

##### A Model of 20S Complex Formation

Quantitation of the components of the 20S particle using a cross-linking approach suggested the presence of three α-SNAP molecules (16). Another study suggested a 3:1 molar ratio based on gel densitometry of fluorescently labeled α-SNAP and SNAP25, although the authors considered the analysis to be semiquantitative due to variability in protein labeling efficiency (17). Moreover, the recent low-resolution reconstruction of the 20S particle from electron microscopy data also suggested the presence of three α-SNAP molecules (18).

Binding of three α-SNAPs to one ternary SNARE complex would require α-SNAP to interact with three different binding sites on the SNARE complex, considering the absence of apparent 3-fold symmetry in the electrostatic potential surface of the SNARE core bundle (Fig. 4). Because we observed only one binding site between α-SNAP and the SNARE complex in our SEC-MALS experiments even at saturating conditions, any additional binding sites would have to be of much weaker affinity.

**FIGURE 5.**
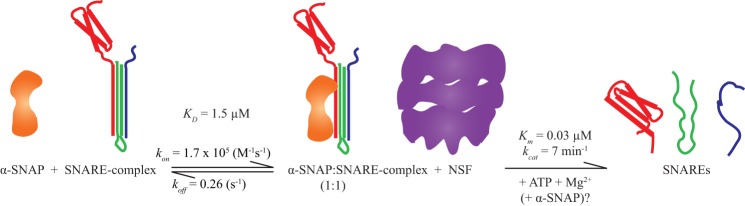
**Model of SNARE complex disassembly.** α-SNAP forms an initial 1:1 complex with the ternary SNARE complex (probably via the t-SNARE components of the complex) with an affinity of 1.5 μm (see Fig. 3). NSF binds to this initial α-SNAP-SNARE complex and then disassembles the ternary SNARE complex through hydrolysis of ATP. Additional α-SNAP molecules may be recruited at this stage or during the disassembly process.

Our data suggest a model (Fig. 5) in which one α-SNAP molecule binds the t-SNARE components of the ternary SNARE complex with medium affinity (*K_D_* = 0.3–1.5 μm). NSF binds the α-SNAP-SNARE complex with high affinity in an arrangement that is not influenced by the VAMP and syntaxin N-terminal domains. The processive mechanism of ATP-driven disassembly by NSF is then initiated (19). At this stage or during the processive disassembly, additional α-SNAP molecules may be recruited. It is possible that two of three putative α-SNAP molecules in the 20S complex do not concurrently contact the SNARE complex. The low resolution of the cryo-EM reconstruction of the 20S complex would allow this possibility (18). If this were the case, subsequent binding and unbinding events of α-SNAP molecules with the SNARE complex could be an important part of the processive disassembly mechanism by NSF.

## Footnotes

2 The abbreviations used are:

VAMP: vesicle-associated membrane protein
TCEP: tris(2-carboxyethyl)phosphine
MALS: multiangle light scattering
SEC: size exclusion chromatography.
